# Effective Electro-Activation Process of Hydrogen Peroxide/Peroxydisulfate Induced by Atomic Hydrogen for Rapid Oxidation of Norfloxacin over the Carbon-Based Pd Nanocatalyst

**DOI:** 10.3390/ijerph191912332

**Published:** 2022-09-28

**Authors:** Ling Yang, Mengmeng Cui, Shiyu Cheng, Shaoqi Zhang, Ying Li, Te Luo, Tianyu Zheng, Hua Li

**Affiliations:** 1 Key Laboratory of Ecology and Environment in Minority Areas, Minzu University of China, National Ethnic Affairs Commission, Beijing 100081, China; 2 College of Life and Environmental Sciences, Minzu University of China, Beijing 100081, China

**Keywords:** persulfate oxidation, atomic hydrogen, organic pollutant, sulfate radical, hydroxyl radical

## Abstract

Peroxydisulfate (PDS) can be activated by electrochemistry, for which using atom H* as an activator is feasibly favorable in theoretical and experimental applications. Studies have shown that atomic H* can cleave the peroxide bond as a single-electron reducing agent in Na_2_S_2_O_8_ to generate SO_4_^•−^, thus achieving the degradation of pollutants. Herein, Pd nanoparticles synthesized by in an in situ solution were dispersed in carbon black and then loaded on carbon felt, called Pd/C@CF, as the cathode for peroxydisulfate activation. This showed an ideal degradation effect on a small electrode (10 mm × 10 mm). Cyclic voltammetry (CV) and linear sweep voltammetry (LSV) tests were taken to verify the significant increase in the yield of the reduction of Na_2_S_2_O_8_ by H*. The degradation experiments and free-radical scavenging experiment confirmed that the atomic H* was the dominant component triggering the activation of PDS to generate SO_4_^•−^. A Pd/C@CF composite electrodes have low pH dependence, high stability and recyclability, etc., which has many potential practical applications in wastewater treatment. In addition, H* can also reduce H_2_O_2_ to •OH by breaking the peroxide bond, so the removal of pollutants by the same amount of H_2_O_2_ and Na_2_S_2_O_8_ under the same conditions is compared, and their application prospects are analyzed and compared.

## 1. Introduction

Nowadays, advanced oxidation processes (AOPs) which produce strongly oxidizing radicals have emerged as a feasible technology [1] for the elimination of various organic pollutants in wastewater [2,3]. After years of development, the types of advanced oxidation technologies have gradually increased, mainly including ozone oxidation, Fenton oxidation, electrochemical oxidation, photocatalytic oxidation and persulfate oxidation [4,5]. Among them, the persulfate oxidation system dominated by SO_4_^•^^−^ is considered a new and efficient advanced oxidation technology developed in recent years [6]. The highly active sulfate radical (SO_4_^•^^−^) is generated by the cleavage of peroxidation bonds in persulfate molecules by energy and electron-transfer reactions. SO_4_^•^^−^ has a remarkable REDOX potential (E^0^ (SO_4_^•^^−^/SO_4_^2^^−^) = +2.60~+3.10 V_NHE_ > E^0^ (•OH/OH^−^) = +1.90~+2.70 V_NHE_) [7]) and a longer half-lifetime (sulfate radical: 30–40 μs; hydroxyl radical: 20 ns [8]). In short, the half-life of a sulfate radical is much longer than that of a hydroxyl radical, which means it has a longer contact time with organic compounds; the standard REDOX potential of SO_4_^•^^−^ is higher than that of a hydroxyl radical, so its oxidation capacity is stronger, and it is easier for the compound to degrade organic matter.

The activator is the key in persulfate (PS) activation system. The basic mechanism of PS activation is the rupture of the O-O bond in the structure [9], including thermal activation [10], alkali activation [11], UV activation [12,13], electrochemical activation [14,15], activated carbon activation [16], ultrasonic activation [17] and transition metal activation [18]. We now concentrate on atomic hydrogen (H*), a single-electron donor that can function as a reducing agent and is a type of intermediate species in the Volmer step—an electrocatalytic water splitting process (Equation (1)) [19,20,21]. Atomic H*, which has been widely used in electrocatalytic hydrodechlorination (EHCD) [22,23] as a reducing agent at the cathode, has recently been found to be a directional activator which catalyzes the conversion of H_2_O_2_ into •OH (Equation (2)) [20,24]. Based on this, we can take it for granted that H* can also be used to effectively transform PS into SO_4_^•^^−^ [25], and relevant studies have proven its feasibility at present (take PDS as an example, Equation (3)) [21]. What is worthy to be mentioned is that the production of atomic H* is independent of the pH value [26]: in acidic conditions, H* is produced by the reduction of H^+^; in neutral or alkaline conditions, H* can result from H_2_O [20]. Besides, the H*/ H^+^ couple has a lower redox potential (E^0^ = −2.1 V vs. RHE) than the Fe^2+^/Fe^3+^ couple (E^0^ = −0.77 V vs. RHE), which indicates H* activation of PS is thermally favorable [27,28].

2H_2_O + 2e^−^ + M → 2(H*)_ads_M + 2OH^−^ (Volmer step) (1)

H* + H_2_O_2_ → •OH + H_2_O (2)

H* + S_2_O_8_^2^^−^ → H^+^ + SO_4_^•^^−^ + SO_4_^2^^−^ (3)

Palladium (Pd), as a well-known cathodic catalyst in the Volmer process [29,30], and is widely recognized for its excellent performance in helping the generation, adsorption, and storage of H* [31,32]. The ideal bonding ability between H* and Pd forms Pd-H* [21,33], inhibiting the reorganization of H* atoms (Heyrovsky and Tafel steps in Equations (4) and (5)) thereby prolonging the lifetime of H* [34,35]. Therefore, H* can effectively activate S_2_O_8_^2^^−^ to SO_4_^•^^−^ in the presence of metal Pd as the catalyst. We chose to disperse Pd effectively in carbon black to create a Pd/C catalyst. In related studies on PS activation, carbon-based materials were found to possess the ability to activate persulfate [36], and the mechanism is shown in the Equations (Equation (6)) [37]. In the process of catalyst synthesis, we carried out pickling for Pd/C catalyst, which would increase the -COOH group in structure and thus promote the activation effect.

(H*)_ads_Pd + H_2_O + e^−^ → Pd + OH^−^ + H_2_ (Heyrovsky step) (4)

(H*)_ads_Pd + (H*)_ads_Pd → 2Pd + H_2_ (Tafel step) (5)

Surface-OOH + S_2_O_8_^2^^−^ → SO_4_^•^^−^ + Surface-OO• + HSO_4_^−^ (6)

Here, we report that Pd nanoparticles were deposited on carbon black in a high dispersion manner and anchored on a conductive support carbon felt. Therefore, carbon felt (CF) is a suitable choice for accommodating Pd/C catalysts due to its excellent electrical conductivity and porous structure. CF has also been shown to activate potential for persulfate activation [36,38]. Experiments show that Pd/C@CF composite material can effectively degrade a variety of organic pollutants when used as a cathode electrode. In addition to H*, PDS can also be activated by carbon black and CF via a nonradical pathway by triggering the electron-transfer process from an electron donor. Various factors affecting the degradation were investigated experimentally, and it was confirmed that H* was electro-generated on the active site of Pd via the H_2_O reduction, and the reduction of PDS in aqueous solution simultaneously generated SO_4_^•^^−^ radicals to cause the degradation of organic matter. Furthermore, recycling and reusability experiments show that the electrocatalytic system has high application potential for the purification of wastewater containing pops. In addition, we also compared the differences in the experiments and electrochemical tests of H* reducing PDS and H_2_O_2_, which also have O-O bonds activated under the same conditions.

## 2. Materials and Methods

### 2.1. Chemicals and Reagents

Carbon fiber (CF, 5 mm × 10 mm × 10 mm) was purchased from Beijing Jinglong Special Carbon Technology Co., Ltd. (Beijing, China) Palladium chloride (PdCl_2_), Vulcan XC-72, sodium carbonate solution (Na_2_CO_3_ 0.5M, pH = 9.4), sodium borohydride (NaBH_4_), sodium sulfate (Na_2_SO_4_), sodium perisulfite (Na_2_S_2_O_8_), tetracycline, norfloxacin, tertbutyl alcohol (TBA), P-benzoquinone (PBQ), methylene blue (MB) and methyl orange (MO) were analytical-grade purity, purchased from Shanghai Macklin Biochemical Co., and were used without further purification. Ultrapure water (Milli-Q ρ = 18.2 MΩcm/25 °C) was used throughout this study.

### 2.2. Synthesis of the Catalyst

The Pd/C@CF catalyst was synthesized by a solution method according to previous studies [39]. In summary, PdCl_2_, water and Vulcan XC-72 carbon were mixed and stirred, and then the pH value adjusted to 8–9 with the Na_2_CO_3_ solution. Then 10 mL aqueous NaBH_4_ solution was added to the suspension, followed by carbon fiber. The resulting suspension was stirred for 4 h at room temperature, then centrifuged and washed three times with distilled water. The resulting catalyst was calcined at 400 °C in N_2_ atmosphere for 2 h. The obtained catalyst was then acid-washed with H_2_SO_4_ for 8 h.

### 2.3. Characterization

The surface morphology and composition of the catalyst were characterized by a transmission electron microscope (TEM) (JEOL JEM-2200FS) and the scanning electron microscope (SEM, Hitachi s-4800), equipped with an energy-dispersive spectrometer (EDX). The X-ray diffraction pattern of the composites with different synthesis conditions were obtained by X-ray diffractometer (XRD, Rigaku SmartLab SE), furnished with a Cu target (K_α radiation, λ = 1.5406 Å) at 40 kV and 40 mA in a 2θ range from 10° to 90°. The data collection step size was 4°, with a collection time of 1 min at each step. MDI Jade 5.0 was used for diffraction peak and crystal phase identification, with JCPDS database as reference. The content of the Pd element in an electrode was detected by inductively coupled plasma–mass spectrometry (ICP-MS, Agilent 7800X).

### 2.4. Electrocatalytic Activity and Electrochemical Analysis

The electrocatalytic activity of the Pd/C@CF electrode was evaluated in degradation of norfloxacin as a probe. All batch experiments were performed in beakers of the same size and were carried out using an electrochemical workstation (CHI-630E, China) equipped with three electrodes. In the degradation system, the Pd/C@CF electrode (10 mm × 10 mm) was used as a working electrode (WE), the Ag/AgCl electrode was used as reference electrode and platinum foil (10 mm × 10 mm) as the counter electrode. A 30-min adsorption was taken to reach the adsorption−desorption equilibrium between the organic pollutant molecules and catalyst electrode in terms of the abasement of 10 mg/L norfloxacin (100 mL), containing 0.1 mol/L Na_2_SO_4_. Specifically, the Pd/C@CF electrode was placed in the norfloxacin solution through the electrode clamp, and the absorption equilibrium was reached after stirring by the rotor for 30 min. After that, the 3 mM PDS was added into the norfloxacin solution, and the catalytic oxidation was started by electrification. The pH of solution was regulated by 0.1 M NaOH or 0.1 M HCl. At predetermined time intervals, 2 mL aliquot was taken out and filtered through a 0.22 μm syringe filter to eliminate particles and mixed with methanol to instantly quench oxidizing radicals. The concentration of norfloxacin was determined by measuring at the characteristic wavelength of 272 nm [40] with an ultraviolet–visible spectrophotometer. The degradation rate of norfloxacin can be obtained by the absorbance of the reaction solution, and the reaction kinetic equation was fitted according to the degradation rate curve [41].

Cyclic voltammetry (CV) and linear sweep voltammetry (LSV) were analyzed on a CHI630E electrochemical workstation to confirm the effect of Pd/C system. The CV and LSV texts were performed in 0.1 M Na_2_SO_4_ with scan rate of 50 mV s^−1^ using an Ag/AgCl electrode as reference electrode, a platinum foil (10 mm × 10 mm) as counter electrode, and glassy carbon electrode (5 mm in diameter) as working electrode (WE), respectively. The preparation of WE strictly ensured that the density of the catalyst was 0.1 mg/cm^2^. First, 1.9625 mg of Pd/C catalyst powder was dispersed in 2 mL of Nafion-containing solution (water: isopropanol: 5% Nafion = 15:4:1), and sonicated for 30 min. Then, 20 μL of catalytic ink was dropped onto the glassy carbon electrode (diameter 5 mm) and dried at room temperature. Before electrochemical analysis, the WE was activated by continuous cyclic voltammetry (CV) cycling between −1.20 V and +0.4 V vs. Ag/AgCl, until a stable voltammogram was obtained in 0.1 M Na_2_SO_4_.

## 3. Results and Discussion

### 3.1. Characterization of Pd/C Catalyst

**Figure 1:**
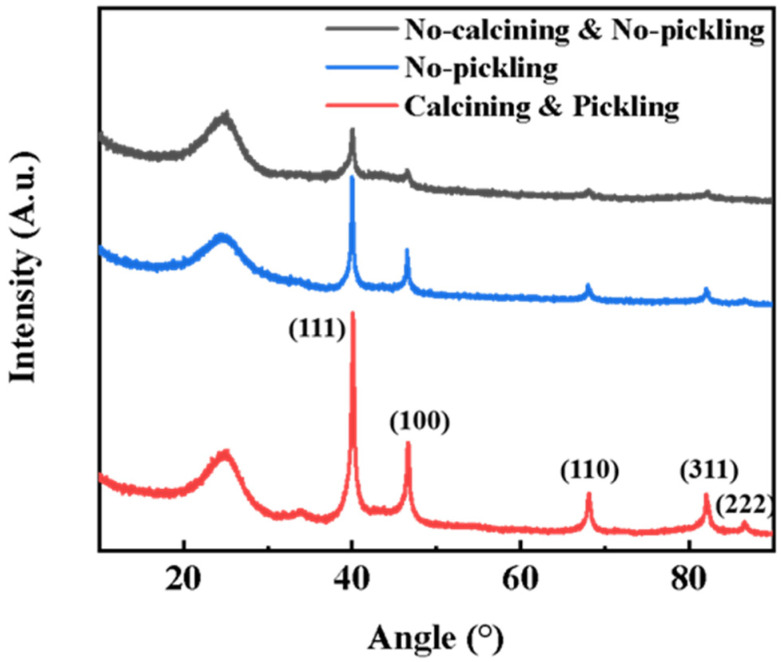
The XRD of Pd/C catalysts under different synthesis conditions.

X-ray diffraction (XRD) was measured to elucidate the chemical properties of the Pd/C catalyst (Figure 1), as well as the properties of the sample during calcination and pickling. The XRD results show that the Pd/C catalyst after calcination and pickling exhibits higher strength peaks, and that the crystals have higher purity. In addition, degradation tests were performed on catalyst electrodes, synthesized under different conditions (Figure S1). The results show that the electrode after calcination and pickling can achieve the best effect. The XRD pattern revealed that the metallic Pd exhibits crystal planes of (111), (100), (110), (311) and (222), which correspond to the diffraction peaks at 2θ = 40.0, 46.5, 67.9, 81.1 and 87.6 (PDF #88-2335) [42], respectively. In addition, previous studies have shown that the Pd (111) lattice can provide ideal active sites to generate atomic H* [43].

**Figure 2:**
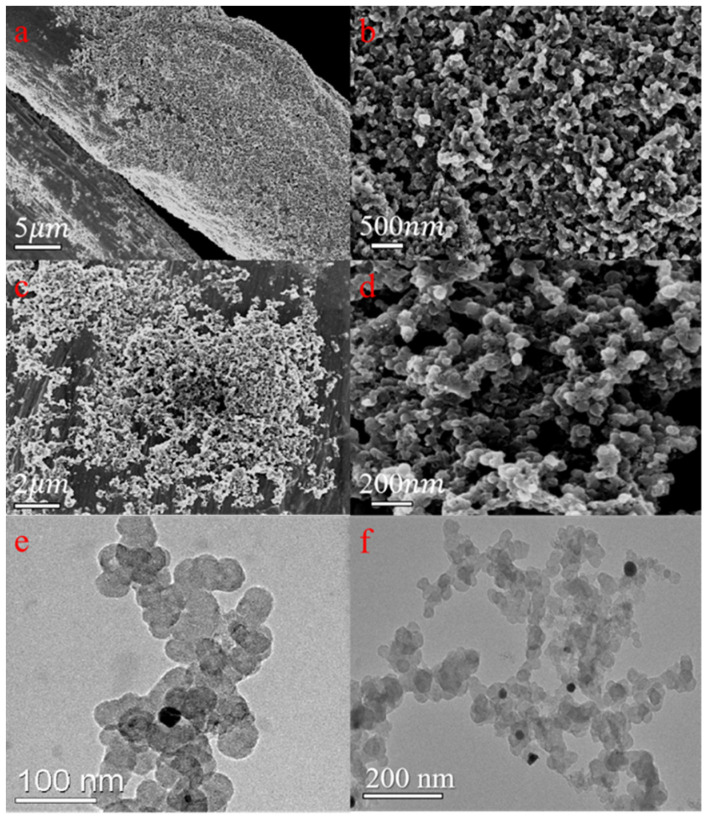
SEM cross-section images (**a**,**b**) of Pd/C@CF, SEM surface images (**c**,**d**) of Pd/C@CF and HRTEM image (**e**,**f**) of Pd/C catalyst.

Figure 2a–d shows the scanning electron microscopy (SEM) image of Pd/C catalyst in situ growing on carbon felt. SEM images of the surface and cross-section presented that catalyst particles were uniformly attached to each carbon fiber of CF. The microstructures of Pd/C catalysts were observed by high-resolution transmission electron microscopy (HRTEM) (Figure 1e,f). The images show Pd NPs with an average diameter of 15–25 nm deposited on the carbon matrix. The SAED pattern (Figure S2) exhibited two crystal planes with a spacing of 0.23 and 0.20 nm, corresponding to Pd (111) and (100) respectively, which can also be expressed in the diffraction peak of XRD. Besides, the loading amount of Pd in the Pd/C@CF (10 mm × 10 mm) was 1.867 g/kg according to the ICP-MS test.

### 3.2. Electrochemical Analysis

**Figure 3:**
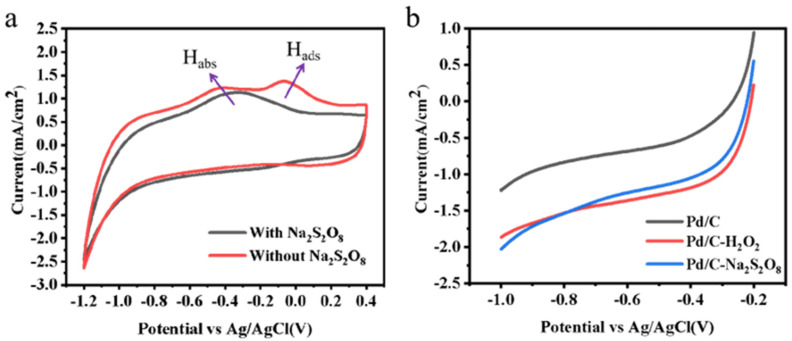
(**a**) CV measurement with Pd/C catalysts in 50 mM Na_2_SO_4_ solution in absence or presence of Na_2_S_2_O_8_ (Na_2_S_2_O_8_, 10 mM; solution pH, 7.0; scanning rate, 50 mV/s); (**b**) LSV of Pd/C catalyst with Na_2_S_2_O_8_ or H_2_O_2_ in 50 mM Na_2_SO_4_ solution (Na_2_S_2_O_8_, 10 mM; H_2_O_2_, 10 mM; solution pH, 7.0; scanning rate, 50 mV/s).

In this work, H* was produced by Pd-catalyzed water-splitting through the Volmer process [44], which continued to activate the peroxide bonds in the PDS. The CV behaviors of Pd/C catalyst were compared by adding the atomic H* scavenger—Na_2_S_2_O_8_—to identify the effect of the H* species. With performing potentials from −1.2 to 0.4 V vs. Ag/AgCl during CV analysis, the generated H* species in the reduction stage were oxidized in the oxidation stage. As shown in Figure 3a, two oxidation peaks in positive scans were exhibited before the addition of Na_2_S_2_O_8_ in the range of −0.1 to 0.1 V and −0.30 to −0.10 V, respectively. With the addition of scavenger, the peak at −0.1 V disappeared and the other peak remained. It can be confirmed from previous studies that the peak at −0.10 V can be assigned to adsorbed H*_ads_, while the peak at −0.30 V refers to the oxidation of absorbed H*_abs_ [45]. Therefore, the activation effect of Na_2_S_2_O_8_ is performed by adsorbed H*_ads_. Therefore, the CV test results indicate that Na_2_S_2_O_8_ is activated by adsorbed H*_ads_.

Meanwhile, the reduction behavior of PDS and H_2_O_2_ on Pd/C was tested by an LSV curve [46]. As can be seen in Figure 3b, the reduction current of Pd/C showed a significant enhancement in cathodic current in comparison with the addition of H_2_O_2_ or PDS, indicating a much-enhanced electrocatalytic activity of Pd/C electrode due to the reduction by H*. As presented in Figure 3b, the LSV curves with H_2_O_2_ and PDS were only slightly different, meaning that H* had almost the same electrocatalytic reduction activity for them. Furthermore, when the voltage is more negative than −0.8 V vs. Ag/AgCl, the cathode current of the PDS system is slightly enhanced, suggesting that H* has lower reduction activity toward H_2_O_2_ than PDS at this time.

### 3.3. Electrocatalytic Activity

**Figure 4:**
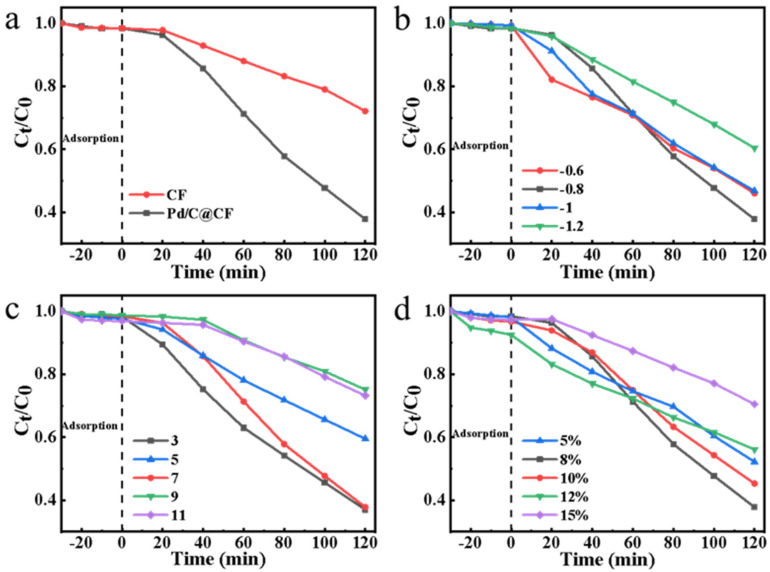
The degradation efficiency of norfloxacin (**a**) with or without Pd/C catalyst on carbon felt (Na_2_S_2_O_8_, 3 mM; norfloxacin, 10 mg/L; pH, 7.0; applied potential, −0.8 V vs. Ag/AgCl; Pd loading, 8 wt%); (**b**) at different applied voltages (Na_2_S_2_O_8_, 3 mM; norfloxacin, 10 mg/L; pH, 7.0; Pd loading, 8 wt%); (**c**) at different initial pH (Na_2_S_2_O_8_, 3 mM; norfloxacin, 10 mg/L; applied potential, −0.8 V vs. Ag/AgCl; Pd loading, 8 wt%); and (**d**) at different Pd loading; (Na_2_S_2_O_8_, 3 mM; norfloxacin, 10 mg/L; pH, 7.0; applied potential, −0.8 V vs. Ag/AgCl).

The effect of the Pd/C@CF catalyst electrode on norfloxacin degradation was studied to determine the electrocatalytic activity of Pd nanoparticles. As shown in Figure 4a, the degradation rate of norfloxacin in 120 min in the presence of Pd/C catalyst was 2.23 times that of carbon felt alone. As expected, the increased chemical activity in the Pd/C@CF system is due to the fact that the resulting atom H* can reduce PDS to SO_4_^•^^−^ radical, the dominant oxidant that destroys organic pollutants.

The effects of the Pd loading, the pH value and the applied voltage on the degradation of norfloxacin were investigated. Figure 4b shows the voltage effect applied: when the voltage increases from −0.6 V vs. Ag/AgCl to −0.8 V vs. Ag/AgCl, the degradation constant increases from 0.006/min to 0.0083/min. However, further increasing the applied voltage to −1.0 V vs. Ag/AgCl reduced the reaction. This phenomenon can be explained as follows: on the one hand, the further increase of applied voltage leads to the generation of more atoms of H*, which results in a series of side reactions with atomic H* (Equations (4) and (5)); on the other hand, a more negative potential leads to excessive production of SO_4_^•^^−^, which also interferes with the reaction (Equations (7) and (8)) [9,47].

SO_4_^•^^−^ + S_2_O_8_^2−^ → S_2_O_8_^•−^+ SO_4_^2−^ (7)

SO_4_^•^^−^ + SO_4_^•^^−^ → S_2_O_8_^2−^ (8)

As for pH effect on the degradation (Figure 4c), the result indicated that norfloxacin degradation efficiency is the highest when pH = 3, and 63% degradation was achieved in 2 h. With the increase of pH value, the degradation efficiency gradually decreases, and the downward trend is more obvious in alkaline conditions. Although previous studies have reported that H_2_O can be used as a precursor for atomic H* in both neutral and alkaline conditions, the slow reaction rate leads to the insufficient generation of the atomic H* for PMS activation. In addition, with the increase of pH, SO_4_^•^^−^ can be quenched to form •OH (Equations (9)–(11)). At alkaline pH, the standard redox potential of •OH is lower than that of SO_4_^•^^−^ [48]. This trend is exacerbated by the increase in pH, resulting in less SO_4_^•^^−^ available for norfloxacin degradation, and thus a reduced oxidative degradation capacity of the entire system [49].

S_2_O_8_^2−^ + H_2_O → 2HSO_4_^•−^ + 0.5O_2_ (9)

S_2_O_8_^2^^−^ + 2H_2_O → 2HSO_4_^•−^ + H_2_O_2_ (10)

SO_4_^•^^−^ + OH^–^ → SO_4_^2−^ + •OH (11)

As shown in Figure 4d, the effects of different Pd loadings on norfloxacin degradation in Pd/C@CF catalytic system are presented here. With the increase of the loading from 5% to 12%, the kinetic degradation constant and the reaction rate first increased then decreased (Figure S3d). As the loading within a certain range increases, more H* will be generated to produce more SO_4_^•^^−^, thus leading to a better degradation of norfloxacin. However, excessive SO_4_^•^^−^ will consume S_2_O_8_^2^^−^, resulting in a reduced catalytic reaction rate (Equations (7) and (8)).

**Figure 5:**
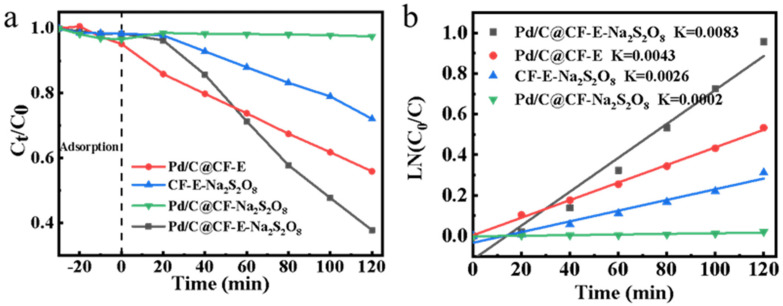
(**a**) Comparison of norfloxacin degradation in various systems; (**b**) the pseudo-first order kinetic rate constants resulting from the different systems shown in (**a**) (electrode area: 10 mm × 10 mm; Na_2_S_2_O_8_, 3 mM; norfloxacin, 10 mg/L; pH, 7.0; applied potential, −0.8 V vs. Ag/AgCl).

In addition, the feasibility of norfloxacin degradation and contribution of each component in the system to the degradation were investigated experimentally (Pd/C@CF-E-PDS system). Figure 5 verifies the factors of catalytic degradation effect of different systems on norfloxacin. As shown in Figure 5b, 62% of norfloxacin in the Pd/C@CF-E-PDS system was rapidly degraded at a first-order kinetic constant of 0.0083/min with a small electrode. The Pd/C@CF-PDS system (no electricity indicates no H* production) and Pd/C@CF-E system have no significant effect on norfloxacin degradation, ruling out the possibility of Pd/C@CF catalyzing PDS activation and atomic H* catalyzing norfloxacin degradation. However, the system without Pd/C (CF-E-PDS system) showed that electrification could not activate PDS well or directly remove pollutants. Clearly, the combination of electrical power and PDS greatly enhances the activity of the system.

**Figure 6:**
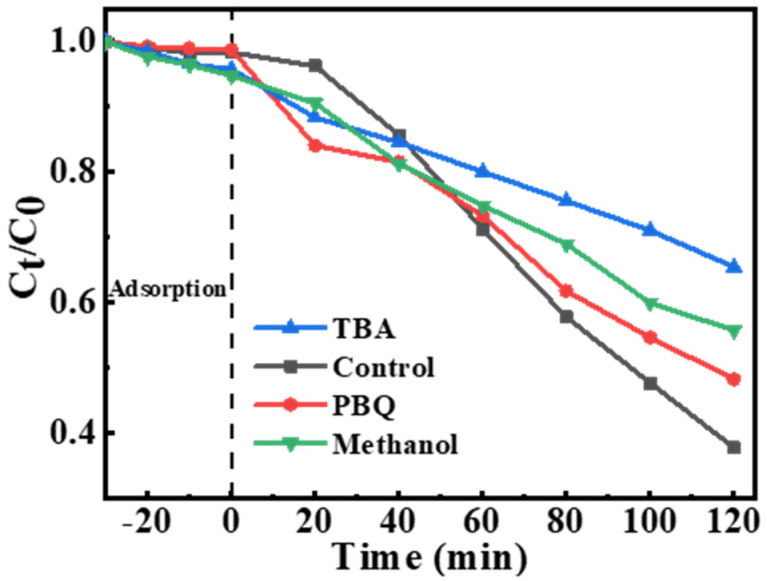
The efficiency of scavengers on the degradation of norfloxacin in Pd/C@CF system (Na_2_S_2_O_8_, 3 mM; norfloxacin, 10 mg/L; TBA, 0.5 M; Methanol, 0.5 M; PBQ, 5 mM; volume of norfloxacin, 100 mL; solution pH, 7.0; applied potential, −0.8 V vs. Ag/AgCl).

Moreover, quenching experiments were performed to confirm the dominant role of free radicals in the system. The quenching experiments with methanol as SO_4_^•^^−^ scavenger further proved the role of SO_4_^•^^−^ in the catalytic system. Tert-butanol (TBA) is an efficient atomic H* scavenger [50]. As shown in Figure 6, the inhibitory effect of TBA on norfloxacin degradation is consistent with the previous reports, indicating that SO_4_^•^^−^ produced by H* is the major oxidizing species in the system [25]. In addition to TBA, p-benzoquinone (PBQ) is an effective •O_2_^−^ scavenge. The system with PBQ had no significant effect on norfloxacin degradation (red line), indicating that •O_2_^−^ was not the dominant species in the system. These results indicated that Pd/C@CF system, with H* as activator and SO_4_^•^^−^ as reactive oxygen radical, could play a good role in the degradation of norfloxacin.

**Figure 7:**
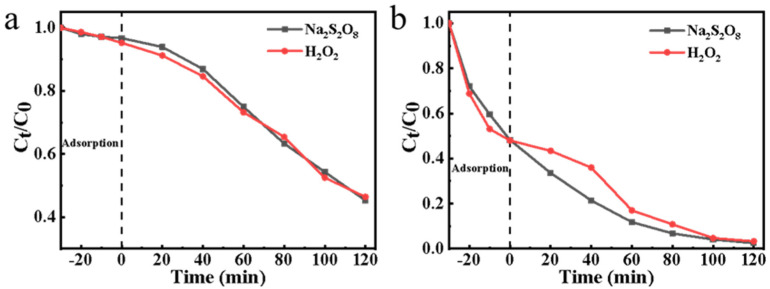
(**a**) Comparison of reduction capacity of atomic hydrogen to H_2_O_2_ and Na_2_S_2_O_8_, and degradation of norfloxacin by different free radicals (H_2_O_2_, 3 mM; Na_2_S_2_O_8_, 3 mM; norfloxacin, 10 mg/L; pH, 7.0; applied potential, −0.8 V vs. Ag/AgCl); (**b**) enlarge the electrode area of (**a**) to 2 × 3 cm^2^ for the degradation experiment (H_2_O_2_, 3 mM; Na_2_S_2_O_8_, 3 mM; norfloxacin, 10 mg/L; pH, 7.0; applied potential, −0.8 V vs. Ag/AgCl).

Herein, the effects of different free radicals were investigated, with equal amounts of H_2_O_2_ and Na_2_S_2_O_8_ under the same conditions. As can be seen from Figure 7a, degradation based on SO_4_^•^^−^ and •OH is basically consistent, which is slightly different from the theoretical redox potential (E^0^ (SO_4_^•^^−^ /SO_4_^2^^−^) is slightly larger than E^0^ (•OH/OH^−^)). This may be due to the obvious ORR performance of carbon felt in the cathode electrode process [51] (catalyzing O_2_ to H_2_O_2_), which can generate additional •OH under the catalytic action of H*. In addition, the PDS system has advantages in the first 100 min due to the higher redox potential and longer life of S_2_O_8_^2−^. When the electrode area was expanded to 2 × 3 cm^2^ (Figure 7b), this phenomenon became more obvious in the amplification experiment. The results in Figure 7b also show that the degradation rate of norfloxacin can reach ~99% by increasing the electrode area appropriately.

### 3.4. Catalyst Reusability and Application Potential

**Figure 8:**
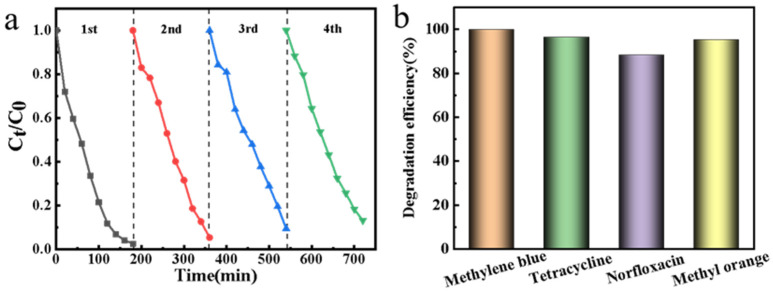
(**a**) Recycling tests of Pd/C@CF for the degradation efficiency of norfloxacin; (**b**) degradation tests of the catalyst on different organic materials (Na_2_S_2_O_8_, 3 mM; norfloxacin, 10 mg/L; solution pH, 7.0; applied potential, −0.8 V vs. Ag/AgCl; volume of pollutants, 200 mL; electrode area: 2 × 3 cm^2^).

For application purposes, the Pd/C@CF system was further evaluated for its recycling potential, as well as its ability to degrade a range of organic pollutants. The electrode with larger electrode area (2 × 3 cm^2^) was selected for performance test, and the results are shown in Figure 8. After four cycles of testing, the degradation efficiency of norfloxacin is relatively stable, indicating a good stability of the catalyst. In addition to norfloxacin, the catalyst was also tested for tetracycline, methylene blue and methyl orange, and the results showed that the catalyst was effective for all three kinds of organic pollutants.

### 3.5. Catalytic Mechanism

**Figure 9:**
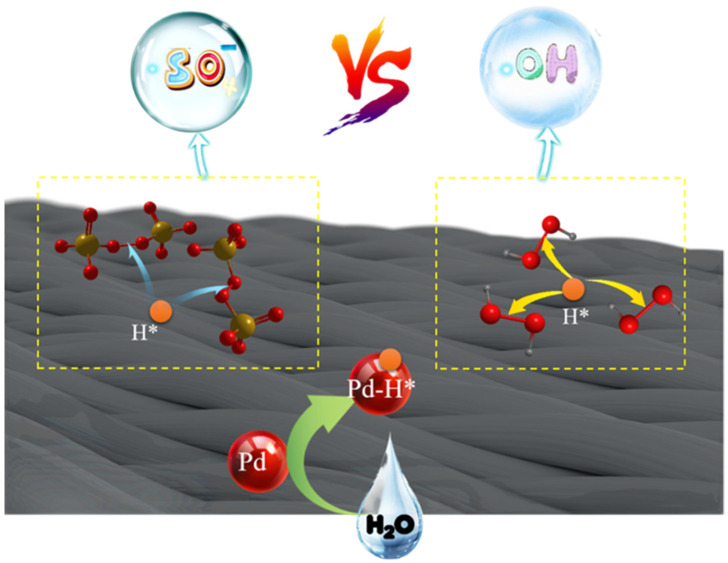
A schematic diagram shows the catalytic mechanism of Pd/C@CF in degradation process of organic pollutants.

Based on our experiment and previous studies, the mechanism of enhanced catalytic activity is assumed as shown in Figure 9. In our electrochemical system, catalyzed by palladium nanoparticles, water molecules first split to form H* atoms through the Volmer process. H* then attacks the O-O bond in the PDS—providing an electron to break it: H* + S_2_O_8_^2^^−^ → H^+^+ SO_4_^•^^−^ + SO_4_^2^^−^ and the highly oxidizing SO_4_^•^^−^ turns organic pollutants into H_2_O and CO_2_. In addition, due to the strong adsorption capacity of carbon felt, the pollutants are degraded after being adsorbed. Therefore, in the process of the degradation reaction, pollutants are constantly consumed on the electrode surface. Residual organic pollutants will also be continuously transferred to the electrode surface, and finally the organic pollutants are completely removed from the system.

We summarize and compare the applications of H* in the reduction of H_2_O_2_ and Na_2_S_2_O_8_. Among them, the advanced oxidation method based on SO_4_^•^^−^ is called AOP-SO_4_^•^^−^; then the advanced oxidation method based on •OH is called AOP-•OH.

(1) Basic performance: E^0^(SO_4_^•^^−^/SO_4_^2^^−^) is slightly larger than E^0^(•OH/OH^−^), and the lifetime of SO_4_^•^^−^ radical is longer than •OH.
(2) Scope of application: AOP-SO_4_^•^^−^ process pH adaptability better than AOP-•OH, and can be applied in a wider range of pH values. This is determined by the reaction mechanism between two free radicals and organic pollutants. AOP-•OH mainly reacts with organic pollutants through the extraction of hydrogen or the addition of hydroxyl [52], while SO_4_^•^^−^ tends to react with organic pollutants through electron transfer [53]. Therefore, SO_4_^•^^−^ has higher activity under neutral and alkaline conditions, and the AOP-SO_4_^•^^−^ system has less stringent pH requirements than the AOP-•OH system.
(3) The practical application: the persulfate used to produce SO_4_^•^^−^ is a solid powder, which is easier to transport and store and more stable than H_2_O_2_, which is more conducive to its widespread use in experiments and engineering. However, H_2_O_2_ can be reduced to O_2_ through an ORR reaction, so the removal of pollutants can be realized without any additional drugs.

Based on the above analysis, we believe that the two advanced oxidation compounds have their own advantages and have good research and application prospects.

## 4. Conclusions

In this study, Pd/C@CF was used as the cathode electrode to construct a system for the activation of PDS by atomic H* and degradation of organic pollutants. Here, we report a catalytic system for the production and storage of H*—Pd/C@CF, the Pd nanoparticles deposited on carbon (Pd/C) in a highly dispersed manner and applying further load on the carbon fiber (CF), by step-dissolved in situ formation of Pd/C@CF composite electrode. Cyclic voltammetry experiments confirm that H* exhibits a high ability to reduce PDS under the action of catalyst, which may be due to the stabilization of H* by Pd nanoparticles. Free-radical scavenging and organic pollutant degradation experiments show that H* reduction of PDS is the key reaction of the system. Various factors affecting the degradation efficiency of norfloxacin, including catalyst load, applied voltage, solution pH value, recovery effect and so on, were explored through the degradation experiment of norfloxacin, and corresponding characterization was carried out. Based on the above experimental results, a hypothesis mechanism for enhancing degradation activity was proposed. The electrode’s repeatability and strong reaction to different pollutants prove its practicability and universality. In addition, the reduction of H_2_O_2_ and Na_2_S_2_O_8_ by H* and the oxidation capacity of SO_4_^•^^−^ and •OH radical are compared, and their application prospects are described.

## Data Availability

Not applicable.

## Supplementary Materials

The following supporting information can be downloaded at: https://www.mdpi.com/article/10.3390/ijerph191912332/s1, Figure S1: Comparison of the degradation efficiencies of methylene blue by employing the Pd/C catalysts synthesized by different methods (Na_2_S_2_O_8_, 3 mM; norfloxacin, 10 mg/L; solution pH, 7.0; applied potential, −0.8 V vs. Ag/AgCl; Pd loading, 8wt%); Figure S2: The SAED pattern of Pd/C catalyst; Figure S3: The apparent rate constant of degradation reaction fitted by pseudo-first-order kinetics equation of Figure 4.
