# Supplementary material for: Effective Electro-Activation Process of Hydrogen Peroxide/Peroxydisulfate Induced by Atomic Hydrogen for Rapid Oxidation of Norfloxacin over the Carbon-Based Pd Nanocatalyst
Source: Int J Environ Res Public Health. 2022 Sep 28;19(19):12332. doi: 10.3390/ijerph191912332 (PMC9566315; doi:10.3390/ijerph191912332)
Supplement: Supplementary file 1 [file ijerph-19-12332-s001.zip › ijerph-1932725-supplementary.pdf]

Support Information

Effective electro-activation process of hydrogen peroxide/peroxydisulfate induced by atomic hydrogen for rapid oxidation of norfloxacin over the carbon based Pd nanocatalyst

Ling Yang [a, b], Mengmeng Cui [a, b], Shiyu Cheng [a, b], Shaoqi Zhang [a, b], Ying Li [a, b], Te Luo [a, b], Tianyu Zheng [a, b], Hua Li [a,b,*]

[a] Key Laboratory of Ecology and Environment in Minority Areas (Minzu University of China), National Ethnic Affairs Commission, Beijing 100081, China

[b] College of Life and Environmental Sciences, Minzu University of China, Beijing 100081, China

* Corresponding author:
E-mail address: lihua@muc.edu.cn.

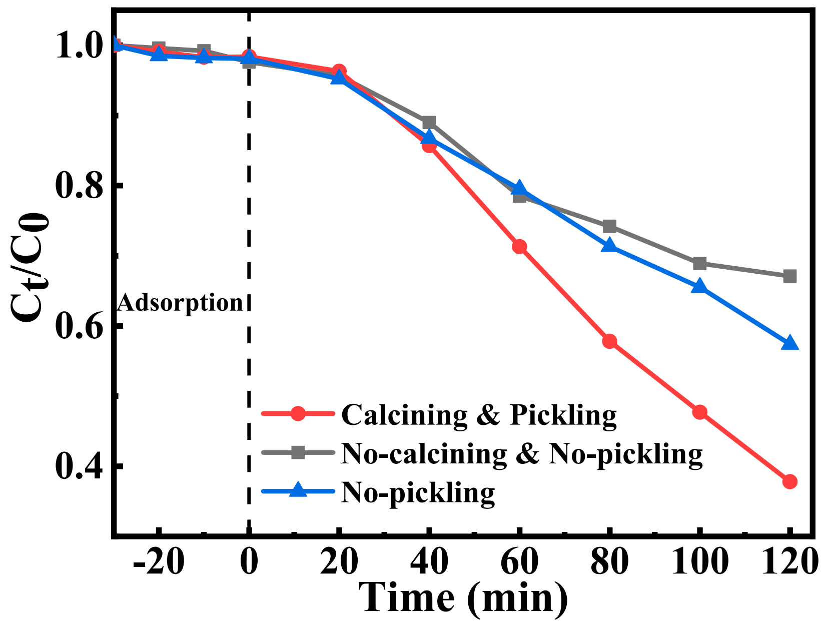

Figure S1 Comparison of the degradation efficiencies of methylene blue by employing the Pd/C catalysts synthesized by different methods ($Na_2S_2O_8$, 3 mM; norfloxacin, 10 mg/L; solution pH, 7.0; applied potential, -0.8 V vs Ag/AgCl; Pd loading, 8wt%)

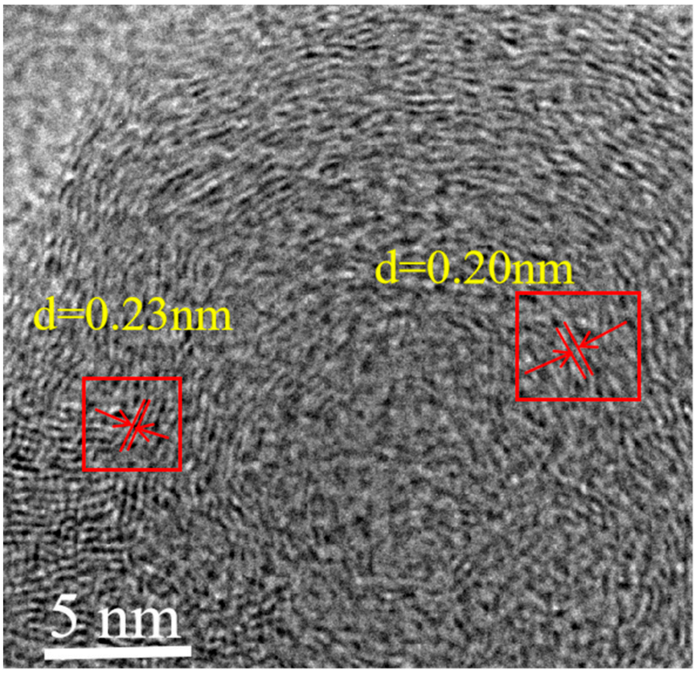

Figure S2 The SAED pattern of Pd/C catalyst.

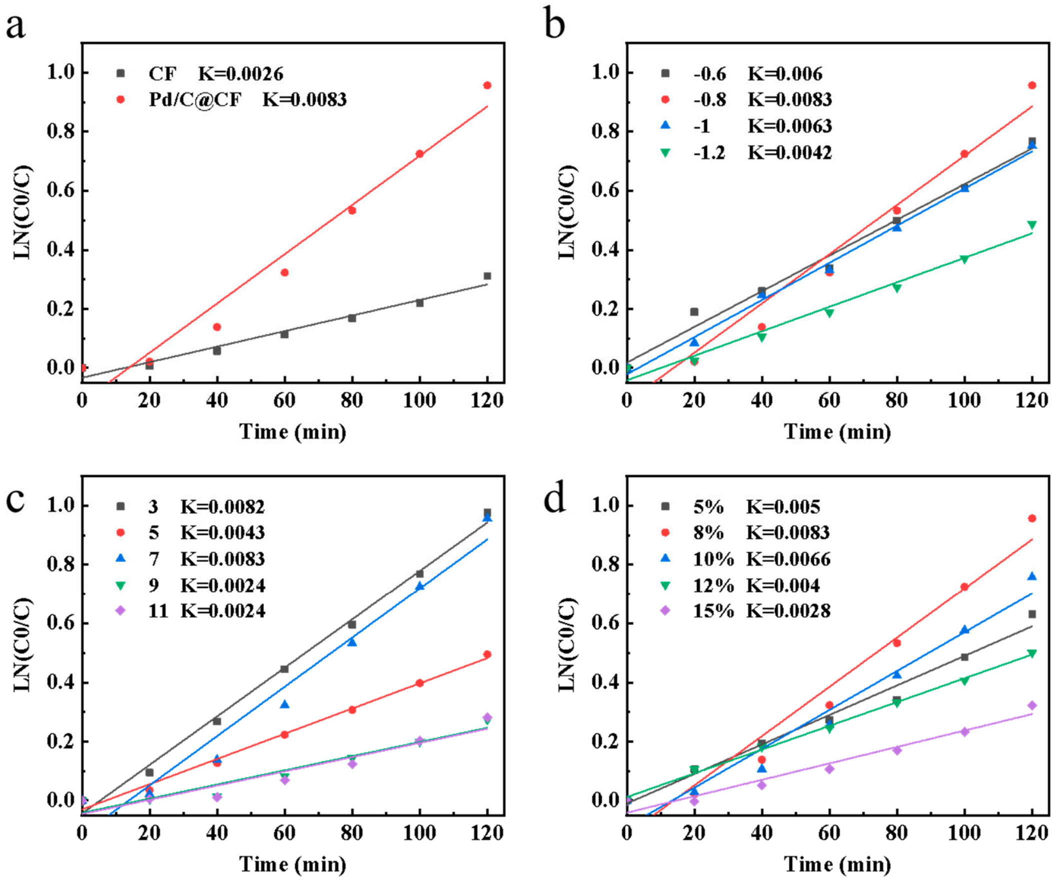

Figure S3 The apparent rate constant of degradation reaction fitted by pseudo-first-order kinetics equation of Figure 4.
